# Causes, Diagnosis, Treatment, and Prognosis of Cardiac Fibrosis: A Systematic Review

**DOI:** 10.7759/cureus.81264

**Published:** 2025-03-27

**Authors:** Hasan A BaniHani, Lana H Khaled, Nada M Al Sharaa, Raghad A Al Saleh, Ahmad K Bin Ghalaita, Ahmad S Bin Sulaiman, Ahmad Holeihel

**Affiliations:** 1 Medicine and Surgery, University of Sharjah, Sharjah, ARE; 2 Family Medicine, University Hospital Sharjah, Sharjah, ARE

**Keywords:** antifibrotic therapies, cardiac biomarker, cardiac fibrosis, fibroblast activation, heart failure

## Abstract

Cardiac fibrosis, characterized by excessive extracellular matrix deposition, contributes to heart failure, arrhythmias, and myocardial dysfunction. Despite advances in understanding its mechanisms, targeted antifibrotic therapies remain limited. This review examines the causes, molecular mechanisms, diagnostic approaches, and therapeutic strategies for cardiac fibrosis. A systematic review of peer-reviewed studies was conducted, focusing on the etiology, diagnosis, treatment, and prognosis of cardiac fibrosis with no specific timeframe. The condition is driven by fibroblast activation, inflammatory pathways, and mechanical stress, with key contributing factors including ischemic heart disease, hypertension, diabetes, and aging. Diagnostic tools such as cardiac magnetic resonance imaging with T1 mapping and biomarkers play a crucial role, with natriuretic peptides offering both diagnostic and prognostic value. Galectin-3 has also shown promise as a prognostic marker. Current therapies, including RAAS inhibitors and beta-blockers, help prevent fibrosis progression but do not reverse established fibrosis. Emerging strategies such as plant-based compounds, gene therapy, fibroblast-targeting vaccines, and stem cell reprogramming show potential in preclinical studies. However, cardiac fibrosis remains a major driver of heart disease progression, and existing treatments remain limited. Major gaps include the lack of validated antifibrotic agents and challenges in translating preclinical findings into clinical applications. Further research is essential to develop effective targeted interventions.

## Introduction and background

Cardiac fibrosis is a complex pathological process characterized by the excessive deposition of extracellular matrix (ECM) proteins, such as collagen, in the myocardial interstitium. This condition is a common response to various myocardial injuries, including myocardial infarction (MI), heart failure, and cardiomyopathies, contributing significantly to the progression of these diseases. Cardiac fibrosis impairs cardiac function, leading to heart failure, arrhythmias, and reduced contractility, making it a critical determinant of cardiovascular disease outcomes. Cardiac fibrosis can be classified into reactive fibrosis, characterized by diffuse ECM deposition without direct cardiomyocyte loss (e.g., hypertension and diabetic cardiomyopathy), and replacement fibrosis, where ECM replaces necrotic cardiomyocytes following MI [1-3].

The process of fibrosis is primarily driven by activated fibroblasts and myofibroblasts, which are influenced by mechanical stress, inflammatory cytokines (such as transforming growth factor-β (TGF-β) and platelet-derived growth factor (PDGF)), and interactions with other cardiac cells, including cardiomyocytes, endothelial cells, and immune cells [1,2]. While fibrosis may initially be reparative - such as following MI, where a scar replaces necrotic tissue - excessive ECM deposition disrupts myocardial architecture, ultimately impairing both systolic and diastolic function [1,3,4].

Two factors play a significant role in ECM deposition. Matrix metalloproteinases (MMPs) are a family of zinc-dependent endopeptidases that degrade various ECM components, facilitating tissue remodeling and repair. However, their dysregulation can contribute to pathological fibrosis. For instance, MMPs such as MMP-2, MMP-3, MMP-8, MMP-11, MMP-12, and MMP-28 exhibit profibrotic roles, while others like MMP-19 have antifibrotic effects. Tissue inhibitors of metalloproteinases (TIMPs), which are endogenous inhibitors of MMPs, are essential for maintaining ECM homeostasis. They regulate MMP activity by binding to them and preventing ECM degradation. The balance between MMPs and TIMPs is critical; an imbalance can lead to either excessive ECM accumulation or degradation [5].

Despite substantial advances in understanding the molecular mechanisms behind cardiac fibrosis, effective and specific therapies remain limited. Current treatment strategies, including renin-angiotensin-aldosterone system (RAAS) inhibitors and beta-blockers, primarily aim to manage the underlying conditions rather than directly targeting the fibrotic process [6,7]. Additionally, diagnostic tools like cardiac magnetic resonance imaging (CMR) and serum biomarkers have limitations in detecting early or diffuse fibrosis, particularly in the absence of visible structural changes [7,8]. These limitations underscore the urgent need for more accurate diagnostic methods and targeted therapies that directly address the fibrotic mechanisms.

This comprehensive review aims to analyze the causes, diagnostic approaches, treatment strategies, and prognostic implications of cardiac fibrosis. We will explore the cellular and molecular mechanisms driving the fibrotic response, review current diagnostic techniques, and examine both pharmacological and nonpharmacological treatment options. Furthermore, we will discuss the challenges in diagnosing and treating cardiac fibrosis and propose potential pathways for future therapeutic advancements.

Cardiac fibrosis remains a critical determinant of heart disease progression, and while our understanding of its mechanisms has advanced, effective treatment options remain an unmet need. This review will contribute valuable insights into the pathophysiology, diagnosis, and management of cardiac fibrosis, with the goal of informing future research and clinical practice in this challenging area of cardiovascular medicine.

## Review

Methodology

Research Question

The research question guiding this review is “What are the key causes and molecular mechanisms driving cardiac fibrosis, how can it be accurately diagnosed, what are the limitations of current treatments, and how can prognosis be improved for patients with cardiac fibrosis?”

This study relied on the Scopus database to collect information related to the causes, diagnostic approaches, treatment strategies, and prognostic implications of cardiac fibrosis. The data was extracted from Scopus on February 5, 2025. Scopus serves as an extensive database offering abstracts, articles, and citations across various disciplines. The search term “cardiac fibrosis” was used to identify pertinent studies, focusing on the title, abstract, and keywords. Additionally, the study included articles containing the terms “etiology”, “causes”, “diagnosis”, “prognosis”, “novel”, and “treatment” in their titles for further analysis.

Search Query

TITLE-ABS-KEY ( "cardiac fibrosis" ) AND ( "causes" OR "etiology" OR "pathogenesis" OR "mechanisms" ) AND ( "diagnosis" OR "diagnostic" OR "biomarkers" OR "imaging" OR "non-invasive" ) AND ( "treatment" OR "therapies" OR "therapeutic strategies" OR "anti-fibrotic" OR "pharmacological" OR "stem cell" ) AND ( "prognosis" OR "outcomes" OR "remodeling" OR "fibrogenesis" ) AND ( "fibroblasts" OR "epigenetics" OR "non-coding RNAs" OR "myofibroblasts" OR "cellular mechanisms" ) LIMIT-TO ( SRCTYPE , "j" ) LIMIT-TO ( PUBSTAGE , "final" ) LIMIT-TO ( SUBJAREA , "MEDI" ) LIMIT-TO ( DOCTYPE , "ar" ) LIMIT-TO ( DOCTYPE , "re" ) LIMIT-TO ( LANGUAGE , "English" )

The search focused on titles, abstracts, and keywords to ensure the inclusion of relevant literature.

Study Selection

**Figure 1:**
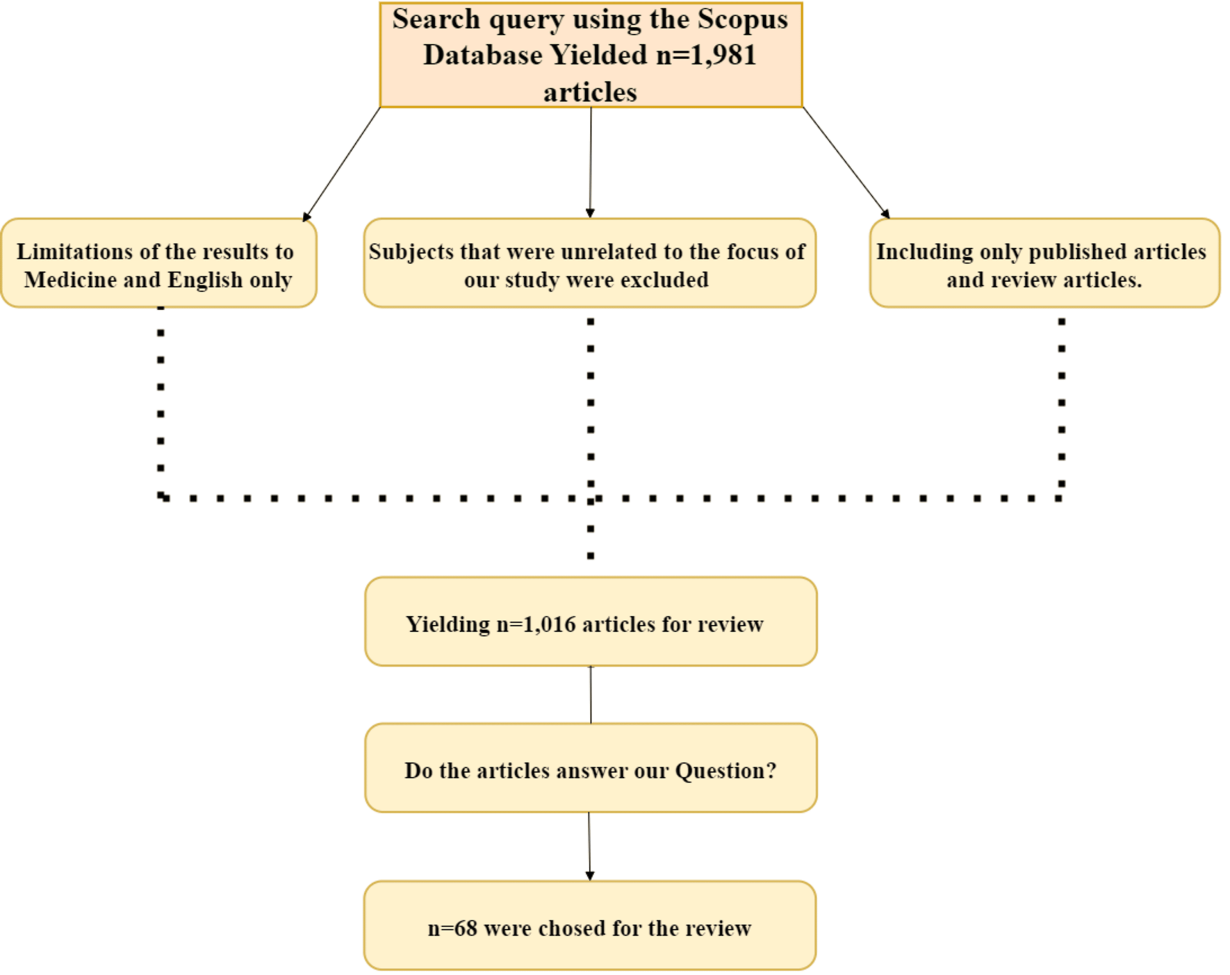
Flowchart depicting the literature search and selection process The initial search yielded 1,981 studies. The selection process involved screening titles and abstracts, followed by assessing articles that met the inclusion criteria for methodological quality and relevance. After resolving discrepancies through discussion, 68 studies were selected for qualitative synthesis.

The subjects unrelated to cardiac fibrosis, such as oncology, neurology, and endocrinology, were excluded from this study. The search was specifically limited to articles that focused on cardiac fibrosis and related topics in cardiovascular research. To ensure the relevance and specificity of the studies, only articles published in peer-reviewed journals were included, excluding non-journal sources like books and conference proceedings. Additionally, only articles written and published in English were considered to maintain consistency in language. The search was not restricted to a particular publication timeframe, as the aim was to assess both the evolution of the understanding of cardiac fibrosis over time and the recent developments in therapeutic strategies. After applying these exclusions and limitations, the final search query in the Scopus database yielded 1981 studies. The authors carefully reviewed these studies for their relevance to the research question. This process led to the exclusion of 1,913 studies, leaving 68 articles to be included in the review (Figure 1).

Molecular and cellular mechanisms

Fibroblast Activation and ECM Deposition

**Figure 2:**
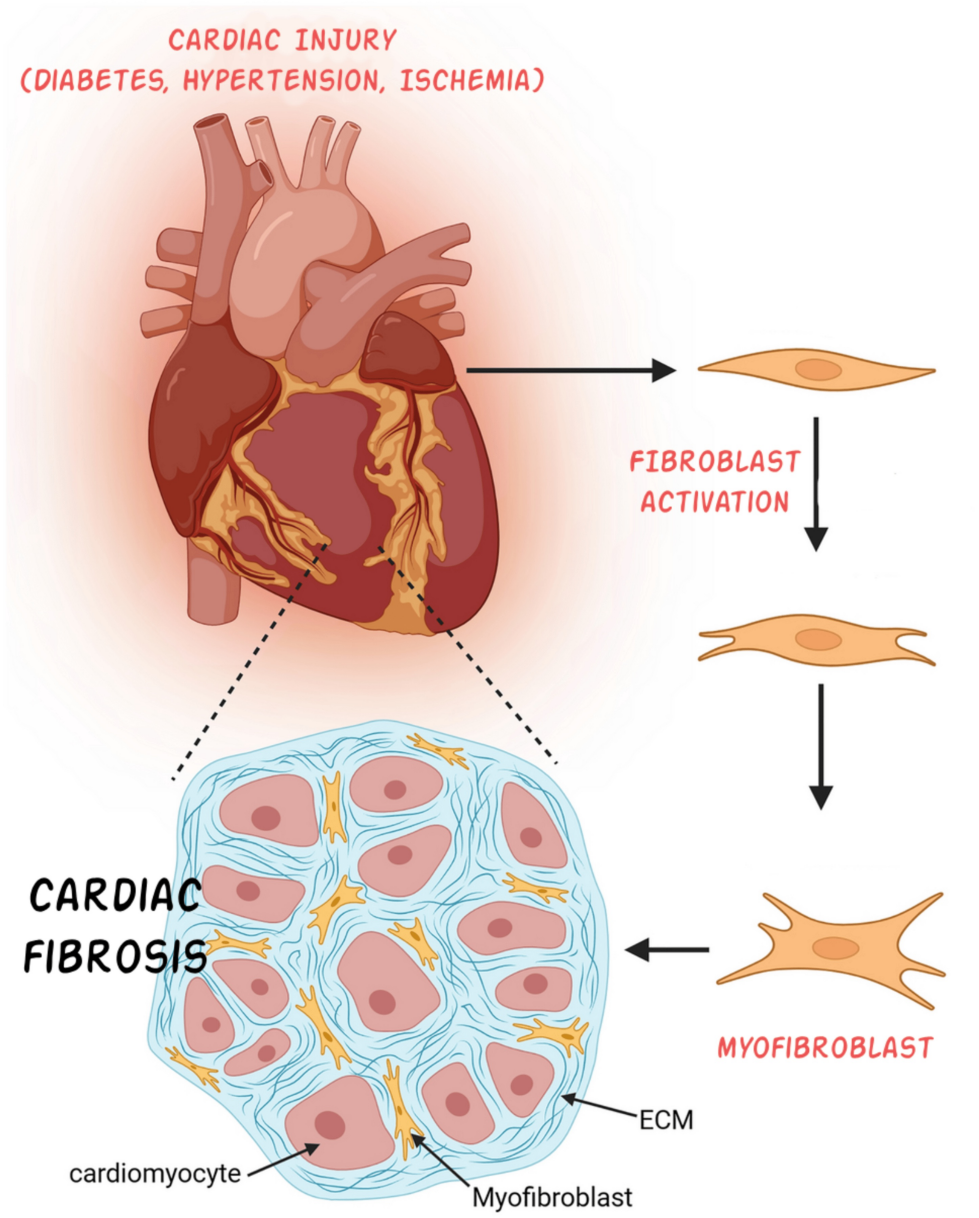
Progression from injury to fibrosis This figure illustrates the key cellular and molecular processes involved in cardiac fibrosis. In response to myocardial injury (e.g., ischemia, hypertension, and diabetes), fibroblasts become activated and differentiate into myofibroblasts, which subsequently secrete excessive ECM [1,9]. ECM, extracellular matrix Image credits: Hasan A. BaniHani

The results of our analysis reveal several key molecular and cellular mechanisms driving cardiac fibrosis. First, cardiac fibroblasts (CFs) play a central role in fibrosis, as they activate and differentiate into myofibroblasts in response to injury, secreting ECM proteins such as collagen (Figure 2). Single-cell RNA sequencing (scRNA-seq) has identified significant heterogeneity among CFs, highlighting distinct subpopulations with specific roles in fibrosis [1,9]. Endocardium-derived fibroblasts, which preferentially proliferate and expand in response to pressure overload, play a significant role in the development of cardiac fibrosis. Their activation is promoted by Wnt signaling, and their specific ablation can alleviate fibrosis and improve heart function [10]. Myofibroblasts are activated fibroblasts that gain contractile function and are critical in promoting replacement fibrosis. They secrete ECM proteins and express smooth muscle α-actin, supporting the necrotic area post-injury. Matrifibrocytes emerge from myofibroblasts during scar maturation. They lose the proliferative ability and smooth muscle α-actin expression but continue to support the mature scar with a unique ECM and tendon gene signature. Immune fibrocytes are involved in immune responses and fibrosis, particularly in hypertension and other cardiovascular diseases. They interact with immune cells and contribute to the fibrotic process [11]. CTHRC1-expressing fibroblasts exhibit a profibrotic signature and are crucial for scar formation post-MI. They are regulated by noncanonical TGF-β signaling and transcription factors like SOX9 [12]. FAP/POSTN fibroblasts are identified in failing human hearts and are driven by IL-1β signaling from CCR2 macrophages. They contribute to myocardial fibrosis and can be targeted to reduce fibrosis and improve cardiac function [13].

Epigenetic Regulation

Epigenetic regulation, including DNA methylation, histone modifications, and noncoding RNAs, also plays a crucial role in CF activation and fibrosis, with targeting epigenetic regulators like DNA methyltransferases (DNMTs) and histone deacetylases (HDACs) showing promise as therapeutic strategies [9,14,15]. DNA methylation and histone modifications regulate fibrosis-related genes by altering gene expression. DNA methylation silences anti-fibrotic genes through CpG island methylation, while histone modifications influence chromatin structure. DNMT3A mediates hypermethylation of the sFRP3 promoter, silencing this Wnt inhibitor and promoting fibroblast activation, whereas DNMT3A knockdown reverses this effect, reducing fibrosis [16]. DNMT1 suppresses SOCS3 via promoter hypermethylation, activating STAT3, which drives fibroblast activation and collagen deposition in diabetic cardiac fibrosis [17]. HDAC3 deacetylates DNMT1, preventing its degradation and leading to SHP-1 suppression via methylation, contributing to cardiac fibrosis and heart failure [18].

Immune-Fibroblast Interactions

Immune-fibroblast interactions, particularly IL-1β signaling between CCR2+ macrophages and CFs, contribute to the emergence of fibrogenic fibroblast subpopulations, and inhibiting this signaling pathway has demonstrated potential in reducing fibrosis and improving cardiac function [13,19]. T-cells and mast cells contribute to cardiac fibrosis through interactions with CFs and the release of pro-fibrotic mediators. CD4+ T-cells, particularly Th1 cells, secrete IFN-γ, which induces fibroblast activation and myofibroblast differentiation. Th1 cells also adhere to fibroblasts via integrin α4, promoting TGF-β production, a key pro-fibrotic cytokine [20]. Mast cells accumulate in the myocardium during cardiac injury and release mediators such as histamine, tryptase, and chymase, which stimulate fibroblast proliferation and collagen synthesis. Mast cell-fibroblast interactions, as seen in transgenic models with TNF overexpression, further enhance fibroblast activation and collagen production, driving fibrosis [21].

​​​​*Mechanotransduction*

CFs sense mechanical stress through mechanosensitive receptors and ion channels, activating fibrogenic cascades that contribute to ECM deposition, particularly under conditions like pressure overload [1,22]. Mechanosensitive receptors and ion channels, including integrins, Piezo1, Piezo2, and TRPV4, play crucial roles in cardiac fibrogenic activation. Integrins (β1 and αv) mediate cell-matrix interactions, activating FAK/Src and TGF-β signaling, which drive fibroblast activation and ECM remodeling [23]. Piezo1, activated by mechanical stress, triggers calcium influx, stimulating p38 MAPK and IL-6 secretion, promoting fibroblast proliferation and ECM production [24,25].

Etiological factors

Ischemic Heart Disease

Cardiac fibrosis is a pathological process resulting from various etiological factors that lead to excessive ECM deposition, ultimately impairing myocardial function. One of the primary causes of cardiac fibrosis is ischemic heart disease, where MI triggers an inflammatory response following cardiomyocyte death. This process results in ECM deposition and scar formation, preventing ventricular rupture but compromising contractile function. Elevated cardiac troponins (cTnI and cTnT) serve as specific biomarkers of myocardial injury during ischemic events. Cardiac fibrosis is a complex process driven by fibroblasts, which can be identified by specific markers such as discoidin domain-containing receptor 2, PDGF receptor-α (PDGFR-α), and the transcription factor 21 (Tcf21). These cells undergo dynamic phenotypic changes in response to myocardial injury. Under stress or infarction, fibroblasts may transform into myofibroblasts, which are distinguished by a well-developed endoplasmic reticulum and the expression of contractile proteins like α-smooth muscle actin (α-SMA). Myofibroblast accumulation has been observed in several pathological conditions, including ischemic cardiomyopathy, MI, hypertension-related heart disease, myocarditis, and alcoholic cardiomyopathy [1]. The identification of fibrotic fibroblasts utilizes specific markers and imaging modalities in both experimental and clinical settings. In experimental research, markers such as discoidin domain-containing receptor 2, PDGFR-α, Tcf21, and α-SMA are employed to study fibroblast activation and differentiation in cardiac fibrosis models. For instance, Tcf21 is essential for CF development, as its absence impairs the epithelial-to-mesenchymal transition necessary for fibroblast progenitor cells [26]. In clinical settings, ischemic fibrosis is detected using specific circulating biomarkers and imaging modalities. Elevated cardiac troponins (cTnI and cTnT) serve as biomarkers of myocardial injury during ischemic events. Imaging techniques such as cardiac MRI with late gadolinium enhancement (LGE) differentiate ischemic fibrosis from nonischemic forms based on scar distribution patterns. Echocardiography with strain imaging assesses myocardial stiffness and dysfunction associated with fibrosis. Additionally, nuclear imaging modalities like PET and SPECT detect metabolic and perfusion abnormalities linked to fibrotic remodeling [27].

Hypertension

Similarly, chronic hypertension induces pressure overload, activating fibroblasts and promoting interstitial fibrosis, which contributes to diastolic dysfunction and heart failure with preserved ejection fraction (HFpEF). Natriuretic peptides, including B-type natriuretic peptide (BNP) and N-terminal pro-BNP (NT-proBNP), correlate with myocardial stretch and serve as biomarkers of hypertensive heart disease [28]. BNP and NT-proBNP are primarily biomarkers of myocardial stretch and heart failure severity. While they reflect increased left ventricular wall stress, their direct correlation with myocardial fibrosis severity is less established [29]. Hypertensive heart disease often leads to both interstitial and perivascular fibrosis. However, hypertensive fibrosis primarily affects the interstitial regions, leading to interstitial fibrosis, which contributes to diastolic dysfunction and HFpEF [11].

Diabetes Mellitus

Diabetes mellitus is another major contributor to cardiac fibrosis through hyperglycemia and insulin resistance, which promote the formation of advanced glycation end products (AGEs), oxidative stress, and activation of profibrotic pathways such as TGF-β1. Biomarkers like galectin-3 and soluble ST2 reflect inflammation and fibrosis in diabetic cardiomyopathy [30]. Myofibroblast accumulation has been observed in several pathological conditions, including MI, ischemic cardiomyopathy, myocarditis, hypertension-related heart disease, and alcoholic cardiomyopathy. However, fibrosis can also occur independently of myofibroblast conversion, as certain fibroblast subsets contribute to collagen deposition and ECM remodeling, as seen in diabetic cardiomyopathy and angiotensin II (AngII)-induced fibrosis [1]. AGEs, oxidative stress, and TGF-β1 activation interact synergistically to accelerate fibrosis by promoting ECM deposition and altering fibroblast function. AGEs induce oxidative stress by increasing reactive oxygen species (ROS) production, which in turn activates TGF-β1, a key profibrotic cytokine. TGF-β1 enhances fibroblast activation, stimulating collagen synthesis and ECM remodeling [31,32]. Notably, fibrosis can occur independently of myofibroblast conversion, as certain fibroblast subsets contribute to collagen deposition without transitioning into contractile myofibroblasts. One such example is the Thy1⁻ fibroblast subset, which has been implicated in diabetic cardiomyopathy and AngII-induced fibrosis, actively participating in ECM remodeling without expressing α-SMA, a hallmark of myofibroblasts [33,34].

Inflammatory and Genetic Disorders

Additionally, inflammatory diseases, including myocarditis and systemic lupus erythematosus (SLE), contribute to myocardial fibrosis through persistent immune activation and fibroblast stimulation, with elevated troponins and CRP indicating myocardial damage and systemic inflammation [35]. Genetic and congenital disorders, such as hypertrophic cardiomyopathy (HCM), also predispose individuals to fibrosis due to mutations in sarcomeric proteins, leading to myocardial disarray and structural remodeling [36]. Immunosuppressive therapy can slow or reduce fibrosis progression in inflammatory diseases by decreasing immune activation and fibroblast stimulation. In conditions like myocarditis and SLE, this therapy helps prevent further myocardial damage and fibrosis [37]. In addition to MYH7 and MYBPC3 mutations, several other genetic mutations are associated with severe fibrosis in various forms of cardiomyopathy. Mutations in TNNT2 (troponin T) and TNNI3 (troponin I) are linked to HCM and are associated with more severe fibrosis and contractile dysfunction. Similarly, mutations in ACTC1 (alpha cardiac muscle actin) are implicated in familial dilated cardiomyopathy (DCM) and contribute to fibrosis in affected individuals. LMNA (lamin A/C) mutations are associated with a range of cardiomyopathies, including DCM, and can lead to fibrosis due to abnormal nuclear structure and function [38,39].

Aging and Environmental Factors

Aging is another key factor associated with increased myocardial fibrosis due to reduced regenerative capacity, chronic low-grade inflammation, and heightened oxidative stress. Elevated levels of natriuretic peptides and galectin-3 are frequently observed in elderly individuals with fibrotic changes [40]. Exposure to toxins and medications, including alcohol, anthracyclines, and recreational drugs, also induces myocardial fibrosis through oxidative stress and direct cardiomyocyte toxicity, often reflected by elevated cardiac troponins [41]. Furthermore, endocrine disorders, such as hyperaldosteronism and hypothyroidism, contribute to fibrotic remodeling through hormonal imbalances that stimulate fibroblast activation and ECM deposition, with increased aldosterone levels playing a significant role in promoting fibrosis [42,43]. The senescence-associated secretory phenotype (SASP) plays a crucial role in age-related myocardial fibrosis by linking cellular senescence with chronic inflammation and ECM remodeling. As cells undergo senescence due to aging, oxidative stress, or DNA damage, they begin secreting pro-inflammatory cytokines such as IL-6, IL-1β, and TNF-α, along with growth factors like TGF-β and VEGF. These factors contribute to persistent low-grade inflammation, fibroblast activation, and excessive ECM deposition, ultimately promoting fibrosis. Additionally, SASP-associated MMPs disrupt ECM homeostasis, leading to pathological remodeling rather than proper tissue repair. In the aging myocardium, this process often results in diffuse fibrosis, primarily affecting the interstitial and perivascular regions rather than being localized to specific myocardial areas. The accumulation of fibrotic tissue contributes to ventricular stiffness, diastolic dysfunction, and an increased risk of HFpEF, a condition commonly observed in elderly individuals [44].

The pathophysiological mechanisms linking fibrosis with elevated cardiac biomarkers highlight the complex interplay between myocardial injury, stress, inflammation, and ECM remodeling. Cardiomyocyte necrosis and apoptosis result in the release of troponins into circulation, while ventricular wall stress and dilation elevate natriuretic peptide levels. Proinflammatory cytokines and immune activation increase CRP and soluble ST2 levels, further exacerbating fibrosis. Additionally, fibroblast activation and collagen deposition are associated with elevated galectin-3 and MMPs, indicating ECM remodeling and fibrotic progression. Understanding these mechanisms is crucial for identifying potential therapeutic targets and improving the clinical management of cardiac fibrosis.

Diagnostic tools

Imaging

Myocardial fibrosis can be assessed using non-imaging methods such as plasma biomarkers, which are nonspecific to the heart, and endomyocardial biopsy, which is the gold standard for histological examination. CMR, however, is broadly used clinically and provides detailed fibrosis assessment through T1 mapping and LGE. T1 mapping recognizes both interstitial and replacement fibrosis by measuring tissue magnetization recovery times, while LGE, the gold standard for imaging fibrosis, identifies focal replacement fibrosis based on delayed gadolinium clearance in diseased tissue. CMR findings have remarkable diagnostic and prognostic implications, impacting clinical plans and monitoring fibrosis advancement or response to treatment over time. Moreover, 18F-fluciclatide, a novel radiotracer targeting αvβ3 integrin, shows potential as a marker of fibrosis and angiogenesis, with increased myocardial uptake in acute but not chronic MI. Novel molecular imaging, such as gallium-68 (68Ga) fibroblast activation protein inhibitor (FAPI) PET, allows direct visualization of active fibrosis. This method differentiates between early fibrosis and advanced scarring and monitors treatment responses. Other tracers, like 18F-fluciclatide and 18F-proline, aim at collagen synthesis and angiogenesis, providing valuable insights into disease activity and progression. These advances improve diagnosis and therapeutic strategies. It is important to know that reactive interstitial fibrosis is generally considered a marker of early disease and is associated with more diffuse forms of myocardial injury, such as hypertension and aortic stenosis. Consequently, interstitial fibrosis is distributed diffusely throughout the myocardium. If the trigger to myocardial injury resolves, then interstitial fibrosis has the potential to reverse, leaving behind a fully functional myocardium. However, if the trigger persists, leading to myocyte cell death, then interstitial fibrosis can progress to replacement fibrosis. F-Fluciclatide has a high uptake in active fibrosis, where myofibroblasts are actively secreting ECM. Hence, it is helpful in recognizing early-stage or progressive fibrosis, which can be seen in ischemic heart disease and hypertensive heart disease. F-proline shows high uptake in stable or end-stage fibrosis, where collagen deposition is more prominent. This can be seen in end-stage HCM and chronic post-MI scar tissue [45]. A case report from the Journal of Cardiology Cases reported on the assessment of myocardial fibrosis using T1 mapping and extracellular volume measurement on CMR for the diagnosis of radiation-induced cardiomyopathy following a cancer diagnosis, and although the case report was specific to a certain secondary cause of cardiomyopathy, it has proved its efficacy especially in high-risk patients [46]. Both [18F]Fluciclatide and [68Ga]FAPI-46 are favorable PET tracers undertaking clinical evaluation. Their incorporation into standard clinical practice awaits further validation and regulatory approval. Hypertensive heart disease, which is treated with antihypertensive RAAS inhibitors such as angiotensin-converting enzyme (ACE) inhibitors or angiotensin receptor blockers (ARBs), is likely to have reversible interstitial fibrosis as these medications aim to reduce myocardial fibrosis and improve left ventricular function. There is currently no proven fibrosis-targeting therapy for HFpEF, despite several clinical studies. The authors stress the need for innovative strategies to lessen fibrosis and enhance the results of HFpEF. There have been a number of studies regarding T1 mapping in radiation-induced fibrosis. However, its significance was not demonstrated, and it does not suggest that high-risk patients should undergo routine CMR screening [1,47,48]. An important limitation of the use of LGE by CMR is that it detects focal fibrosis and not diffuse fibrosis, as it works on detecting high-intensity areas in CMR. Newer techniques such as T1 mapping have shown promise in detecting diffuse fibrosis and may provide additional valuable prognostic information in patients. So, as T1 mapping offers diagnostic advantages, mainly for early-stage disease, it has limitations. There can be an overlap in T1 values across different cardiomyopathies and with normal values. Abnormalities in T1 and ECV must be explained and interpreted within the clinical context, and they can be influenced by various disease processes [49-51].

Biomarkers

**Figure 3:**
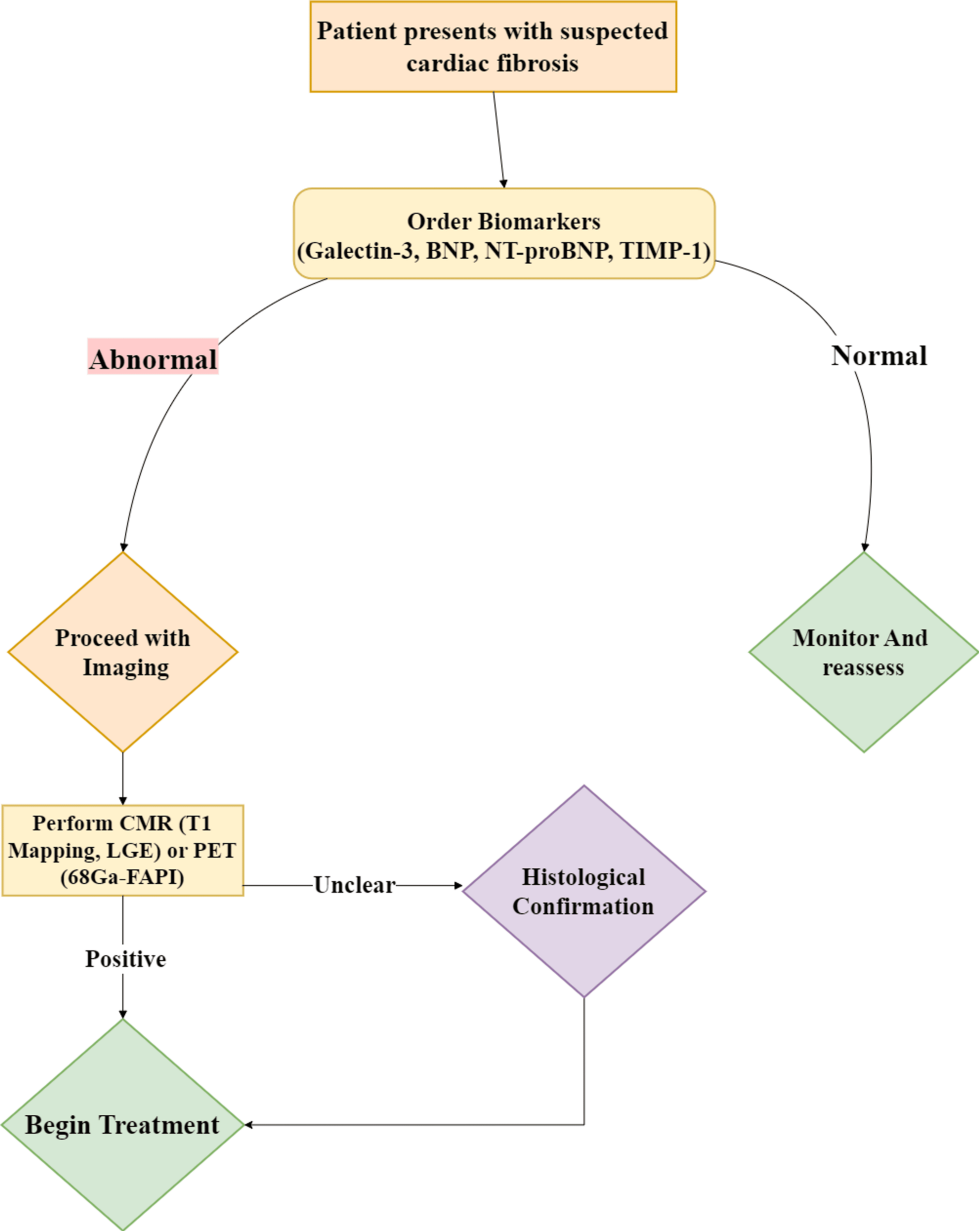
Diagnostic algorithm for cardiac fibrosis Patients with suspected cardiac fibrosis are evaluated based on symptoms (e.g., heart failure and arrhythmias) and risk factors (e.g., hypertension, diabetes, and aging) before undergoing biomarker testing. Blood biomarkers such as Galectin-3, BNP, NT-proBNP, and TIMP-1 are measured. If results are normal, fibrosis is unlikely, and the patient is monitored. If abnormal, further evaluation is required. Imaging studies, including CMR with T1 mapping/LGE or PET scans (68Ga-FAPI), are then used to assess fibrosis severity. Positive findings lead to treatment consideration, while inconclusive cases necessitate additional testing. For uncertain cases, histological confirmation via endomyocardial biopsy is performed as the gold standard to confirm fibrosis [45,46,52,53]. BNP, B-type natriuretic peptide; CMR, cardiac magnetic resonance imaging; NT-proBNP, N-terminal pro-B-type natriuretic peptide; TIMP-1, tissue inhibitor of metalloproteinases-1 Image credits: Hasan A. BaniHani

ProBNP is a highly cardiac-specific biomarker as it is released due to myocyte stretching. Galectin-3, a moderate cardiac biomarker, is involved in cardiac fibrosis as it is produced by activated macrophages during the inflammatory process [48]. A study aimed at investigating whether 99mTc-radiolabeled FAPI (99mTc-HFAPi) imaging can detect early myocardial fibrosis in hypertensive hearts found that fibrosis could be identified before structural and functional myocardial changes. Using an experimental model with spontaneously hypertensive rats that underwent 99mTc-HFAPi imaging and echocardiography, researchers observed early fibrotic processes. This was further supported by measuring RNA and protein expression levels of FAP and collagen I using quantitative PCR and western blot. Autoradiography and histology of the left ventricle confirmed that fibrotic changes were detectable before any structural or functional alterations [52]. According to a study investigating TIMP-1 as a marker of left ventricular diastolic dysfunction and fibrosis in hypertension, patients with hypertension exhibited higher levels of TIMP-1, CITP, and PICP compared to healthy individuals. This suggests increased collagen synthesis, degradation, and inhibition. TIMP-1 was associated with impaired cardiac relaxation and was particularly elevated in individuals with diastolic dysfunction. Moreover, diastolic dysfunction was highly probable in individuals whose TIMP-1 levels exceeded a certain threshold, indicating that TIMP-1 may serve as a reliable noninvasive marker of myocardial fibrosis in hypertension [53]. A flowchart outlining the diagnostic tools used to reach this diagnosis is presented in Figure 3.

Current and emerging treatments

Pharmacological Treatments

RAAS inhibitors, including ACE inhibitors (ACEIs), ARBs, and aldosterone antagonists, block the RAAS to reduce vasoconstriction, sodium retention, and fibrosis. AngII promotes fibroblast proliferation and collagen synthesis, while aldosterone induces inflammation and fibrosis. By inhibiting these pathways, RAAS inhibitors help mitigate cardiac remodeling. To assess their effectiveness, in vitro studies exposed neonatal rat CFs to AngII or TGF-β1, followed by telmisartan treatment, which has been shown to inhibit collagen synthesis and metabolic imbalance in CFs under the influence of AngII [54]. RAAS inhibitors significantly reduced fibrosis by inhibiting collagen synthesis and decreasing fibrotic markers (collagen 1, connective tissue growth factor, and α-SMA). Clinical trials showed improved heart function and reduced heart failure progression. While exact success rates vary, evidence suggests RAAS inhibitors substantially reduce fibrosis and improve clinical outcomes in a significant proportion of patients [55,56]. To address the incomplete blockade of aldosterone by ACEIs and ARBs, mineralocorticoid receptor antagonists such as spironolactone and eplerenone are often added to the treatment regimen. These drugs directly block the effects of aldosterone, providing additional cardiovascular and renal protection [57]. Clinical trials indicate that ACEIs are superior to ARBs in reducing cardiovascular and all-cause mortality, likely due to their anti-fibrotic effects. A large-scale study (KAMIR-NIH) of 12,481 AMI patients found that ACEIs significantly reduced cardiovascular mortality (1.9% vs. 3.5%, HR: 0.562, P < 0.01) and all-cause mortality (2.9% vs. 5.7%, HR: 0.567, P < 0.01) compared to ARBs. This may be due to ACEIs’ ability to inhibit Ang II formation and enhance bradykinin activity, promoting vasodilation and antifibrotic pathways, whereas ARBs only block Ang II at the AT1 receptor [58].

Plant-Based Compounds

Berberine mitigates cardiac fibrosis in diabetic cardiomyopathy by downregulating IGF-1R expression, reducing fibroblast proliferation and collagen deposition. It also inhibits MMP-2 and MMP-9, preventing ECM degradation and myocardial stiffness. Berberine improved glucose tolerance, reduced hyperglycemia, and significantly inhibited fibrosis in rats. Treated rats showed reduced collagen deposition and improved cardiac function. While clinical success rates remain unknown, berberine presents a promising antifibrotic therapy, warranting further human trials for validation [59]. Resveratrol acts by inhibiting the TGF-β/Smad3 pathway, which is central to fibrotic progression, and by activating AMPK and Sirt1 signaling, promoting autophagy, and reducing inflammation. Additionally, resveratrol suppresses NF-κB, lowering pro-inflammatory cytokines like IL-1β, IL-6, and TNF-α. In animal models, resveratrol significantly improved cardiac function, with an increase in ejection fraction by 15-25%, and reduced oxidative stress by decreasing ROS and malondialdehyde. It also decreased fibrosis in models of AngII-induced cardiac hypertrophy by 30%. Effectiveness studies indicate resveratrol reduces fibrosis by 50% in animal studies, improving survival rates by 20-40% in heart failure models. Gallic acid targets the TGF-β/Smad3 signaling pathway, inhibiting fibroblast activation and reducing ECM deposition. It also decreases oxidative stress by targeting NADPH oxidase and enhancing nitric oxide bioavailability, protecting the myocardium. In preclinical studies, gallic acid led to a 40-60% reduction in fibrosis markers like collagen I and α-SMA. It also showed protective effects against Ang II-induced cardiac hypertrophy by reducing cell size and hypertrophic markers. Gallic acid’s dose-dependent effects, particularly at 100 mg/kg/day, resulted in a near-complete reversal of fibrotic changes and a 20-35% improvement in ejection fraction, making it a promising therapeutic. Quercetin enhances mitochondrial function by targeting the SIRT3/PARP-1 pathway, reducing oxidative stress, and inhibiting fibroblast proliferation. It also suppresses hypertrophic markers like ANP, BNP, and β-MHC, which are critical for cardiac remodeling. In vitro studies demonstrated a 50-70% reduction in fibrosis markers such as α-SMA and collagen types I and III. In animal models, quercetin restored mitochondrial membrane potential, reduced hypertrophy, and inhibited the AKT/AMPK pathway. The compound improved cardiac function by 25-45% and significantly reduced fibrotic areas by 40-60%, highlighting its broad potential in treating cardiac fibrosis and hypertrophy. Curcumin inhibits fibrosis through the downregulation of the mTOR pathway and suppression of MMPs (MMP-2 and MMP-9), which degrade ECM components. It also reduces inflammation by lowering levels of TNF-α and IL-6. In animal models of transverse aortic constriction (TAC)-induced cardiac hypertrophy, curcumin reduced fibrosis by 50-60% and improved cardiac ejection fraction by 20-30%. Histological studies revealed significant reductions in collagen deposition in the myocardium. Curcumin’s anti-fibrotic and anti-inflammatory properties make it a promising therapeutic agent for cardiac fibrosis, with preclinical results showing substantial improvements in heart function and fibrotic markers [60].

Enzyme and Signaling Pathway Inhibitors

PDE10A regulates cardiac fibrosis and hypertrophy by promoting fibroblast proliferation and ECM production in response to hypertrophic and profibrotic stimuli. Inhibition of PDE10A increases cAMP and cGMP levels, reducing fibrosis and myocardial dysfunction. Researchers used pharmacological (TP-10, a PDE10A inhibitor) and genetic (PDE10A knockout) approaches. Both methods significantly reduced fibrosis and hypertrophy, improving cardiac function. RNA sequencing further identified PDE10A-regulated pathways linked to cardiac dysfunction. While clinical success rates remain unknown, promising preclinical results suggest PDE10A inhibitors could be effective in heart failure treatment, warranting further clinical trials [61]. TNAP promotes cardiac fibrosis post-MI by activating TGF-β1/Smads and ERK1/2 pathways, leading to fibroblast differentiation, migration, and excessive collagen deposition, which impairs cardiac function. Targeting TNAP inhibits these pathways, reducing fibrosis and improving myocardial recovery. In vivo studies using adenovirus-mediated TNAP knockdown (adv-shTNAP) in MI mouse models showed significant improvements in LVEF and FS and reduced myocardial fibrosis, while TNAP overexpression worsened cardiac function. In vitro studies confirmed that TNAP inhibition suppressed fibroblast activation. Clinically, higher serum TNAP levels correlated with fibrosis biomarkers and increased mortality risk. Preclinical interventions showed up to a 50% reduction in collagen deposition, demonstrating TNAP’s therapeutic potential [62]. The FAP vaccine targets FAP-expressing myofibroblasts to reduce cardiac fibrosis through antibody-dependent cell cytotoxicity and complement-dependent cytotoxicity. It also downregulates fibrosis-related genes (Tgfb1, Col1a1, and Acta2), limiting ECM deposition. In a study using mice under chronic cardiac stress, the vaccine significantly reduced fibrosis and FAP-positive cells by 45%. While LVEF improvements were modest, fibrosis markers decreased by 60%. Unlike CAR-T therapy, the vaccine posed no toxicity or inflammatory risks, offering a safer, cost-effective, and broadly applicable fibrosis treatment [63]. HIPK2 inhibition prevents cardiac fibrosis by downregulating EGR3 and CLEC4D, key regulators of pathological remodeling, and suppressing Smad3 phosphorylation, which limits fibroblast proliferation. The study used pharmacological and genetic methods, administering HIPK2 inhibitors (tBID, PKI1H) in a TAC mouse model and employing HIPK2 knockout (HIPK2⁻/⁻) mice. Results showed significant improvements in cardiac function, including increased fractional shortening (FS), reduced left ventricular mass, and decreased left ventricular internal diameter in systole and left ventricular posterior wall thickness in diastole. Fibrosis was markedly reduced, with lower collagen deposition and fibrosis marker expression. Capillary density increased, enhancing myocardial perfusion. HIPK2 inhibition demonstrated substantial fibrosis reduction, positioning it as a promising therapeutic strategy [64].

Gene Therapy

Gene therapy using AAV9-mediated knockdown of EGR3 and CLEC4D, along with peptide-based therapy CCDC80tide and miR-30d overexpression. Mechanisms include disrupting hypertrophic signaling, inhibiting STAT3 to reduce inflammation, and downregulating MAP4K4 and GRP78 to suppress NFAT signaling. Methods included pharmacological inhibitors (tBID and PKI1H), genetic knockouts, AAV9-mediated shRNA delivery, and molecular analyses like qRT-PCR and Western blot. HIPK2 inhibition improved FS and reduced fibrosis. Gene therapy for EGR3 and CLEC4D decreased fibrosis markers, while miR-30d therapy reduced fibrotic markers by 50-70%, showing strong therapeutic potential for cardiac fibrosis reversal [65].

Stem Cell and Lineage Reprogramming

Stem cell and lineage reprogramming involves the conversion of CFs into cardiomyocyte-like cells, aiming to reduce scar tissue and promote myocardial regeneration. This process leverages transcription factors, small molecules, and epigenetic modifications to induce cellular reprogramming, ultimately restoring cardiac function. While still in the experimental phase, preclinical studies have demonstrated promising results that showed a reduction in fibrosis and restored cardiac function (current reprogramming efficiency ranges from 12% to 60% depending on methods used), suggesting that this approach could offer a viable therapeutic strategy for cardiac repair in the future. However, further studies are needed to optimize efficiency, ensure functional integration of reprogrammed cells, and address potential safety concerns before clinical translation [66].

To sum up, the treatment of cardiac fibrosis involves a diverse range of approaches, from pharmacological interventions to regenerative medicine. RAAS inhibitors and plant-based compounds offer significant antifibrotic benefits, while enzyme inhibitors and gene therapy provide targeted molecular interventions. Stem cell therapy and lineage reprogramming hold promise for long-term cardiac regeneration. Although these treatments show potential, further research is necessary to optimize their efficacy, ensure safety, and transition from preclinical to clinical application.

Experimental models and animal models of cardiac fibrosis

To investigate the progression of cardiac fibrosis and evaluate therapeutic interventions, various experimental models, including animal models, in vitro studies, and microfluidic platforms, have been employed. Among the animal models, the MI model, induced by ligation of the left anterior descending coronary artery in mice, has been widely used to study replacement fibrosis, which occurs following cardiomyocyte death. This model provides insights into fibroblast activation, ECM remodeling, and potential therapeutic interventions. The TAC model is another widely utilized approach that simulates pressure overload in mice, leading to interstitial and perivascular fibrosis, enabling the study of fibroblast-mediated fibrotic progression in response to chronic mechanical stress [67]. Additionally, the AngII infusion model, which involves continuous AngII administration, has been instrumental in studying hypertensive cardiac fibrosis in mice and has revealed sex-specific differences in fibrotic progression, including the identification of fibroblast subpopulations such as Fibroblast-Cilp, a key fibrogenic driver [37]. Furthermore, the hypertensive cardiac fibrosis model in rats has been employed to examine fibrosis resulting from systemic hypertension, highlighting the significant roles of TGF-β, RAAS, and inflammatory cytokines in driving fibrosis progression [1]. In in vitro studies, primary cultures of CFs and myofibroblasts have been used to investigate ECM secretion, including collagen types I and III, fibronectin, and periostin, and to evaluate the effects of therapeutic agents such as TGF-β inhibitors and ARBs [68]. Furthermore, scRNA-seq and proteomics approaches have been instrumental in identifying fibroblast diversity, distinguishing reparative vs. pro-fibrotic fibroblast subpopulations, and demonstrating the role of epigenetic regulation (DNA methylation and histone modifications) in fibroblast activation [8]. Beyond traditional models, an innovative organism-on-a-chip platform has been introduced to bridge the gap between in vitro and in vivo fibrosis studies. This system utilizes a microfluidic device implanted in embryonated chicken eggs, allowing real-time imaging of fibrotic progression and demonstrating a significant increase in cellular density and vascularization within 24 hours post-implantation. Additionally, this model enables the quantification of ECM properties and collagen organization, presenting a viable alternative to animal testing [68].

​​Discussion 

Cardiac fibrosis remains a major contributor to heart failure, yet current treatments largely focus on managing underlying conditions rather than directly targeting fibrosis itself. The fibrotic process is driven by fibroblast activation, excessive ECM deposition, and chronic inflammation, leading to structural and functional cardiac impairment. While traditional therapies, such as RAAS inhibitors, provide some benefits, they do not reverse established fibrosis. Advances in imaging and biomarker discovery have improved early detection, yet challenges remain in specificity and accessibility. Emerging therapies, including plant-derived compounds, gene therapy, and fibroblast-targeted interventions, have demonstrated antifibrotic potential in preclinical studies. However, translating these findings into clinical practice requires further validation through large-scale trials. Additionally, regenerative approaches, such as stem cell therapy and fibroblast reprogramming, hold promise for myocardial repair but face challenges regarding integration, immune response, and long-term stability.

Cardiac fibrosis is a critical factor in heart failure progression, yet current treatments primarily manage underlying conditions rather than directly targeting fibrosis itself [1]. The process is driven by activated fibroblasts that transform into myofibroblasts, leading to excessive ECM deposition and myocardial stiffening, ultimately impairing heart function [9]. Advanced imaging techniques, such as CMR with T1 mapping and PET-based tracers, offer more precise detection of fibrosis, while biomarkers like troponins, natriuretic peptides, and galectin-3 help assess disease severity [45]. Traditional therapies, including RAAS inhibitors, have shown benefits in slowing fibrosis progression but do not reverse established fibrosis [54]. Emerging treatments, such as plant-derived compounds (resveratrol, quercetin, and curcumin), gene therapy, and fibroblast-targeted vaccines, demonstrate promising antifibrotic effects in preclinical studies but require further clinical validation [60,63]. To advance treatment, research must focus on developing fibrosis-specific therapies, exploring regenerative strategies like stem cell reprogramming, and integrating precision medicine approaches to improve patient outcomes [67]. While significant advancements have been made in understanding cardiac fibrosis, several limitations and challenges remain. Current therapies, such as RAAS inhibitors and beta-blockers, primarily manage underlying conditions rather than targeting fibrosis directly [6]. Biomarkers lack specificity, as elevated levels of BNP, galectin-3, and TIMP-1 may occur in various cardiac and systemic diseases, making them less reliable for fibrosis-specific diagnosis [53]. Advanced imaging modalities such as CMR and PET scanning provide high-resolution fibrosis assessment, but their cost and limited accessibility restrict widespread clinical application [45]. Additionally, while plant-based therapies and gene therapies have demonstrated antifibrotic effects in preclinical studies, large-scale clinical trials are lacking, hindering their translation into clinical practice [59]. Furthermore, stem cell and lineage reprogramming strategies remain experimental, with challenges related to cell integration, immune response, and long-term stability [66]. There are also contradictory findings regarding the role of fibroblast subpopulations in fibrosis progression. While some studies suggest certain fibroblast subsets drive fibrosis, others indicate fibroblast plasticity allows for the potential reversal of fibrosis under specific conditions [9]. Standardization of methodologies is necessary to resolve inconsistencies in fibroblast classification and treatment responses.

Future research should prioritize fibrosis-specific therapeutic targets, precision medicine approaches, and improved diagnostic tools to enhance patient outcomes. Standardizing methodologies in fibroblast classification and treatment responses will be crucial for advancing fibrosis management and developing effective, targeted interventions.

## Conclusions

Cardiac fibrosis remains a major contributor to heart disease and heart failure, yet effective therapies remain limited. While advances in imaging and biomarker detection have improved early diagnosis, current treatments focus on managing underlying conditions rather than directly targeting fibrosis. Promising experimental therapies, including gene therapy, antifibrotic agents, and regenerative medicine, offer hope for future treatment breakthroughs. Continued research is essential to develop targeted interventions that halt or reverse fibrosis progression and improve patient outcomes.

## References

[1] Frangogiannis NG (2021). Cardiac fibrosis. Cardiovasc Res.

[2] Frangogiannis NG (2019). Cardiac fibrosis: cell biological mechanisms, molecular pathways and therapeutic opportunities. Mol Aspects Med.

[3] Kong P, Christia P, Frangogiannis NG (2014). The pathogenesis of cardiac fibrosis. Cell Mol Life Sci.

[4] Kurose H (2021). Cardiac fibrosis and fibroblasts. Cells.

[5] Chuliá-Peris L, Carreres-Rey C, Gabasa M, Alcaraz J, Carretero J, Pereda J (2022). Matrix metalloproteinases and their inhibitors in pulmonary fibrosis: EMMPRIN/CD147 comes into play. Int J Mol Sci.

[6] Fang L, Murphy AJ, Dart AM (2017). A clinical perspective of anti-fibrotic therapies for cardiovascular disease. Front Pharmacol.

[7] Ravassa S, López B, Treibel TA (2023). Cardiac fibrosis in heart failure: focus on non-invasive diagnosis and emerging therapeutic strategies. Mol Aspects Med.

[8] Cojan-Minzat BO, Zlibut A, Agoston-Coldea L (2021). Non-ischemic dilated cardiomyopathy and cardiac fibrosis. Heart Fail Rev.

[9] Aguado-Alvaro LP, Garitano N, Pelacho B (2024). Fibroblast diversity and epigenetic regulation in cardiac fibrosis. Int J Mol Sci.

[10] Han M, Liu Z, Liu L (2023). Dual genetic tracing reveals a unique fibroblast subpopulation modulating cardiac fibrosis. Nat Genet.

[11] Torimoto K, Elliott K, Nakayama Y, Yanagisawa H, Eguchi S (2024). Cardiac and perivascular myofibroblasts, matrifibrocytes, and immune fibrocytes in hypertension; commonalities and differences with other cardiovascular diseases. Cardiovasc Res.

[12] Ruiz-Villalba A, Romero JP, Hernández SC (2020). Single-cell RNA sequencing analysis reveals a crucial role for CTHRC1 (collagen triple helix repeat containing 1) cardiac fibroblasts after myocardial infarction. Circulation.

[13] Amrute JM, Luo X, Penna V (2024). Targeting immune-fibroblast cell communication in heart failure. Nature.

[14] Lin LC, Liu ZY, Tu B (2024). Epigenetic signatures in cardiac fibrosis: focusing on noncoding RNA regulators as the gatekeepers of cardiac fibroblast identity. Int J Biol Macromol.

[15] Shao J, Liu J, Zuo S (2022). Roles of epigenetics in cardiac fibroblast activation and fibrosis. Cells.

[16] Jiang SX, Zhou ZY, Tu B (2024). Epigenetic regulation of mitochondrial fission and cardiac fibrosis via sFRP3 promoter methylation. Cell Mol Life Sci.

[17] Tao H, Shi P, Zhao XD, Xuan HY, Gong WH, Ding XS (2021). DNMT1 deregulation of SOCS3 axis drives cardiac fibroblast activation in diabetic cardiac fibrosis. J Cell Physiol.

[18] Wang YY, Gao B, Yang Y (2022). Histone deacetylase 3 suppresses the expression of SHP-1 via deacetylation of DNMT1 to promote heart failure. Life Sci.

[19] Hoque MM, Gbadegoye JO, Hassan FO, Raafat A, Lebeche D (2024). Cardiac fibrogenesis: an immuno-metabolic perspective. Front Physiol.

[20] Learmonth M, Corker A, Dasgupta S, DeLeon-Pennell KY (2023). Regulation of cardiac fibroblasts by lymphocytes after a myocardial infarction: playing in the major league. Am J Physiol Heart Circ Physiol.

[21] Zhang W, Chancey AL, Tzeng HP (2011). The development of myocardial fibrosis in transgenic mice with targeted overexpression of tumor necrosis factor requires mast cell-fibroblast interactions. Circulation.

[22] Ferrari S, Pesce M (2019). Cell-based mechanosensation, epigenetics, and non-coding RNAs in progression of cardiac fibrosis. Int J Mol Sci.

[23] Stewart L, Turner NA (2021). Channelling the force to reprogram the matrix: mechanosensitive ion channels in cardiac fibroblasts. Cells.

[24] Blythe NM, Muraki K, Ludlow MJ (2019). Mechanically activated Piezo1 channels of cardiac fibroblasts stimulate p38 mitogen-activated protein kinase activity and interleukin-6 secretion. J Biol Chem.

[25] Braidotti N, Chen SN, Long CS, Cojoc D, Sbaizero O (2022). Piezo1 channel as a potential target for hindering cardiac fibrotic remodeling. Int J Mol Sci.

[26] Venugopal H, Hanna A, Humeres C, Frangogiannis NG (2022). Properties and functions of fibroblasts and myofibroblasts in myocardial infarction. Cells.

[27] Hanneman K (2022). The clinical significance of cardiac MRI late gadolinium enhancement in hypertrophic cardiomyopathy. Radiology.

[28] Schimmel K, Ichimura K, Reddy S, Haddad F, Spiekerkoetter E (2022). Cardiac fibrosis in the pressure overloaded left and right ventricle as a therapeutic target. Front Cardiovasc Med.

[29] Miyaji Y, Iwanaga Y, Nakamura T, Yasuda M, Kawamura T, Miyazaki S (2016). Interrelationship between the myocardial mass, fibrosis, BNP, and clinical outcomes in hypertrophic cardiomyopathy. Intern Med.

[30] Tuleta I, Frangogiannis NG (2021). Fibrosis of the diabetic heart: clinical significance, molecular mechanisms, and therapeutic opportunities. Adv Drug Deliv Rev.

[31] Chen Y, Meng Z, Li Y, Liu S, Hu P, Luo E (2024). Advanced glycation end products and reactive oxygen species: uncovering the potential role of ferroptosis in diabetic complications. Mol Med.

[32] Richter K, Kietzmann T (2016). Reactive oxygen species and fibrosis: further evidence of a significant liaison. Cell Tissue Res.

[33] Schnee J, Hsueh W (2000). Angiotensin II, adhesion, and cardiac fibrosis. Cardiovasc Res.

[34] Abebayehu D, Pfaff BN, Bingham GC (2024). A Thy-1-negative immunofibroblast population emerges as a key determinant of fibrotic outcomes to biomaterials. Sci Adv.

[35] Myhr KA, Zinglersen AH, Pecini R, Jacobsen S (2024). Myocardial fibrosis associates with lupus anticoagulant in patients with systemic lupus erythematosus. Int J Cardiovasc Imaging.

[36] Correale M, Santoro F, Magrì D (2023). Fibrosis-specific biomarkers and interstitial fibrosis in hypertrophic cardiomyopathy. Kardiol Pol.

[37] Frustaci A, Chimenti C (2015). Immunosuppressive therapy in myocarditis. Circ J.

[38] Keyt LK, Duran JM, Bui QM (2022). Thin filament cardiomyopathies: a review of genetics, disease mechanisms, and emerging therapeutics. Front Cardiovasc Med.

[39] Hasselberg NE, Haland TF, Saberniak J (2018). Lamin A/C cardiomyopathy: young onset, high penetrance, and frequent need for heart transplantation. Eur Heart J.

[40] Biernacka A, Frangogiannis NG (2011). Aging and cardiac fibrosis. Aging Dis.

[41] Thomas SA (2017). Chemotherapy agents that cause cardiotoxicity. US Pharm.

[42] Azibani F, Benard L, Schlossarek S (2012). Aldosterone inhibits antifibrotic factors in mouse hypertensive heart. Hypertension.

[43] Azibani F, Fazal L, Chatziantoniou C, Samuel JL, Delcayre C (2013). Aldosterone mediates cardiac fibrosis in the setting of hypertension. Curr Hypertens Rep.

[44] Childs BG, Gluscevic M, Baker DJ, Laberge RM, Marquess D, Dananberg J, van Deursen JM (2017). Senescent cells: an emerging target for diseases of ageing. Nat Rev Drug Discov.

[45] Barton AK, Tzolos E, Bing R (2023). Emerging molecular imaging targets and tools for myocardial fibrosis detection. Eur Heart J Cardiovasc Imaging.

[46] Mukai-Yatagai N, Haruki N, Kinugasa Y (2018). Assessment of myocardial fibrosis using T1-mapping and extracellular volume measurement on cardiac magnetic resonance imaging for the diagnosis of radiation-induced cardiomyopathy. J Cardiol Cases.

[47] Bentestuen M, Ladekarl M, Knudsen A, Zacho HD (2024). Diagnostic accuracy and clinical value of [68Ga]Ga-FAPI-46 PET/CT for staging patients with ovarian cancer: study protocol for a prospective clinical trial. BMC Cancer.

[48] Sweeney M, Corden B, Cook SA (2020). Targeting cardiac fibrosis in heart failure with preserved ejection fraction: mirage or miracle?. EMBO Mol Med.

[49] Haaf P, Garg P, Messroghli DR, Broadbent DA, Greenwood JP, Plein S (2016). Cardiac T1 mapping and extracellular volume (ECV) in clinical practice: a comprehensive review. J Cardiovasc Magn Reson.

[50] Sano M, Satoh H, Suwa K (2015). Characteristics and clinical relevance of late gadolinium enhancement in cardiac magnetic resonance in patients with systemic sclerosis. Heart Vessels.

[51] Kuruvilla S, Adenaw N, Katwal AB, Lipinski MJ, Kramer CM, Salerno M (2014). Late gadolinium enhancement on cardiac magnetic resonance predicts adverse cardiovascular outcomes in nonischemic cardiomyopathy: a systematic review and meta-analysis. Circ Cardiovasc Imaging.

[52] Xie B, Li L, Lin M (2023). 99mTc-HFAPi imaging identifies early myocardial fibrosis in the hypertensive heart. J Hypertens.

[53] Lindsay MM, Maxwell P, Dunn FG (2002). TIMP-1: a marker of left ventricular diastolic dysfunction and fibrosis in hypertension. Hypertension.

[54] Pan J, Liu M, Su H, Hu H, Chen H, Ma L (2024). Pharmacological inhibition of P-Rex1/Rac1 axis blocked angiotensin II-induced cardiac fibrosis. Cardiovasc Drugs Ther.

[55] Hegarová M, Málek I (2013). Possibilities of influencing the myocardial remodeling. Cor Va.

[56] Zhang Y, Zhao NA, Wang JK (2015). Telmisartan inhibited angiotensin II-induced collagen metabolic imbalance without directly targeting TGF-β 1/Smad signaling pathway in cardiac fibroblasts. Minerva Cardioangiol.

[57] Verdugo FJ, Montellano FA, Carreño JE, Marusic ET (2014). Mineralocorticoid receptor antagonists and therapeutic strategies of cardiovascular damage [Article in Spanish]. Rev Med Chil.

[58] Choi IS, Park IB, Lee K (2018). Angiotensin-converting enzyme inhibitors provide better long-term survival benefits to patients with AMI than angiotensin II receptor blockers after survival hospital discharge. J Cardiovasc Pharmacol Ther.

[59] Li G, Xing W, Zhang M (2018). Antifibrotic cardioprotection of berberine via downregulating myocardial IGF-1 receptor-regulated MMP-2/MMP-9 expression in diabetic rats. Am J Physiol Heart Circ Physiol.

[60] Stankovic S, Mutavdzin Krneta S, Djuric D, Milosevic V, Milenkovic D (2025). Plant polyphenols as heart's best friends: from health properties, to cellular effects, to molecular mechanisms of action. Int J Mol Sci.

[61] Chen S, Zhang Y, Lighthouse JK (2020). A novel role of cyclic nucleotide phosphodiesterase 10A in pathological cardiac remodeling and dysfunction. Circulation.

[62] Cheng X, Wang L, Wen X (2021). TNAP is a novel regulator of cardiac fibrosis after myocardial infarction by mediating TGF-β/Smads and ERK1/2 signaling pathways. EBioMedicine.

[63] Yoshida S, Hayashi H, Kawahara T (2025). A vaccine against fibroblast activation protein improves murine cardiac fibrosis by preventing the accumulation of myofibroblasts. Circ Res.

[64] Zhou Q, Meng D, Li F (2022). Inhibition of HIPK2 protects stress-induced pathological cardiac remodeling. EBioMedicine.

[65] Li J, Sha Z, Zhu X (2022). Targeting miR-30d reverses pathological cardiac hypertrophy. EBioMedicine.

[66] Zeng N, Tang W, Wu Y, Fan H, Xie S, Cao N (2023). Harnessing stem cell and lineage reprogramming technology to treat cardiac fibrosis. Cell Regen.

[67] Raimondi MT (2024). Organisms-on-a-chip. EPJ Web of Conferences.

[68] McLellan MA, Skelly DA, Dona MS (2020). High-resolution transcriptomic profiling of the heart during chronic stress reveals cellular drivers of cardiac fibrosis and hypertrophy. Circulation.

